# Deep condolences to Professor Fuyu Yang

**DOI:** 10.52601/bpr.2023.230901

**Published:** 2023-02-28

**Authors:** Editorial Board of Biophysics Reports

**Affiliations:** 1

Professor Fuyu Yang, an academician of the Chinese Academy of Sciences and outstanding biochemist, passed away in Beijing on January 5, 2023.

**Figure 1:**
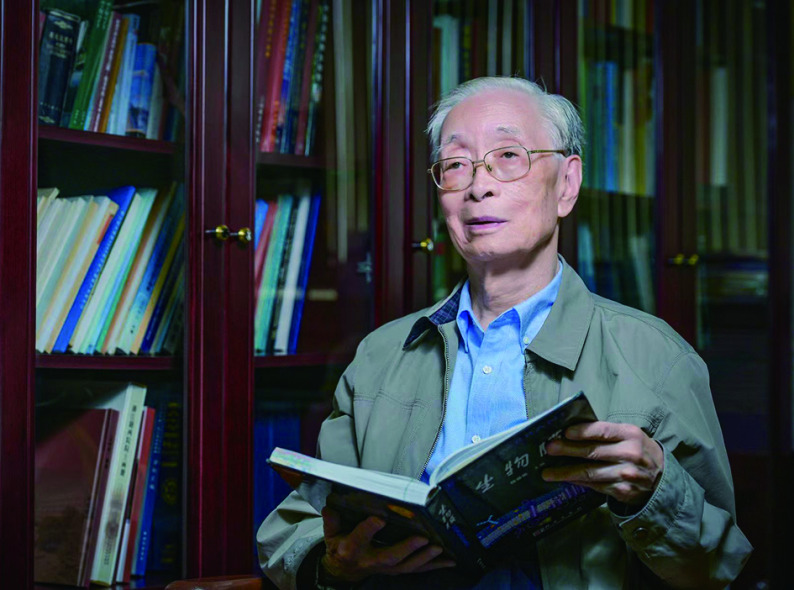
Professor Fuyu Yang

Professor Yang was born in Shanghai in October 1927 and was originally from Zhenhai, Ningbo, Zhejiang Province. He graduated from the Department of Chemistry of Zhejiang University in 1950, and then started his work at the Institute of Experimental Biology, the Chinese Academy of Sciences (Shanghai). Under the guidance of Professor Shizhang Bei, a famous Chinese biophysicist, and other senior scientists, he quickly became an active and excellent young scientist. In 1958, he was selected by the state to go to study in the Department of Biology of Moscow University in the Soviet Union, was engaged in mitochondrial oxidative phosphorylation research, and obtained his PhD degree in biology from Moscow University in May 1960.

**Figure 2:**
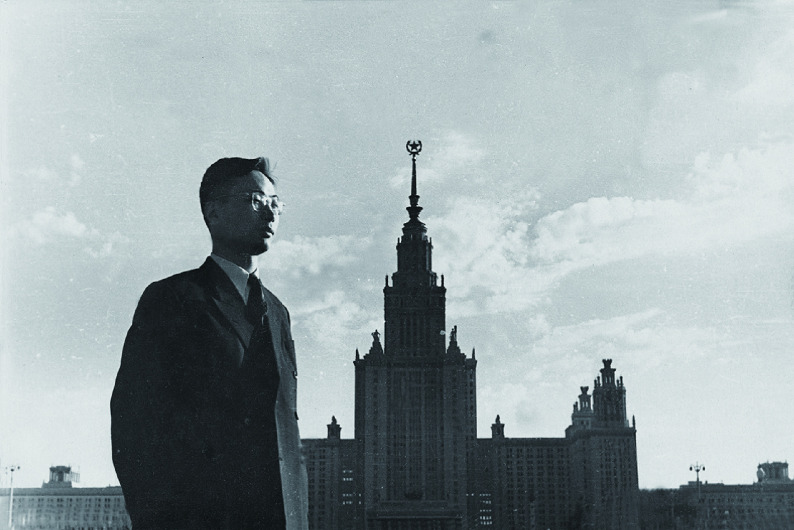
Professor Fuyu Yang in front of Moscow University

After returning to China, Professor Yang joined the Institute of Biophysics of the Chinese Academy of Sciences in Beijing, set up a research team on mitochondrial structure and function, and led the group to study the effects of ionizing radiation on the mitochondrial oxidative phosphorylation function. Since then, he had established the important research direction of “structure and function of biomembrane”, for which he strived all his lifetime. He was praised by Academician Shizhang Bei as the first person for biomembrane research in China. In 1991, Professor Yang was elected an academician of the Chinese Academy of Sciences.

Professor Yang carried out long-term systematic and in-depth original research around lipid–protein interactions, as well as their regulatory mechanism. He proposed a new mechanism that magnesium ions affect the structure and activity of mitochondrial ATPase by changing the fluidity of membrane lipids, providing a clear example of how the physical state (fluidity) of membrane lipids affects the conformation and function of membrane proteins, revealing a new mechanism that the physical state of membrane lipids regulates Ca-ATPase and G-protein coupled transmembrane signal transduction pathways, and found that chymotrypsin is not only a digestive enzyme, but also mediates the lysosome–mitochondrial pathway which induces apoptosis.

In addition, Professor Yang attached great importance to the combination of basic theoretical research and practical application. He led his team to participate in many major national scientific research projects, such as the biological effects of previous Chinese nuclear tests and the impact of long-term low-dose ionizing radiation on the hematopoietic and reproductive systems of rhesus monkeys, summed up biochemical indicators for the early diagnosis of chronic radiation sickness, laying the foundation for nuclear safety. He also used the “uniformity complementarity method” to predict the hybridization advantages of crops, providing a scientific basis for increasing agricultural production. And he led his team to investigate the etiology of Keshan disease, and put forward the view that Keshan disease is a “myocardial mitochondrial disease”.

Professor Yang successfully used interdisciplinary methods to carry out research at different levels, and the research results were pioneering and original. He published more than 200 papers in academic journals and two monographs including *Biomembrane*. He had a monumental and tangible impact on biomembrane globally and was widely recognized. He successively won the National Natural Science Award, the CAS Natural Science Award, the Ho Leung Ho Lee Prize, and other important awards.

Professor Yang was very enthusiastic about the training of young talents. As the winner of the third Shizhang Bei Award initiated by the Biophysical Society of China, he donated CNY 300,000 to the Biophysical Society of China in 2018 for commending the subsequent winners of the Shizhang Bei Award for Young Biophysicists, showed his ardent expectations for young scientific and technological researchers in the field of biophysics.

**Figure 3:**
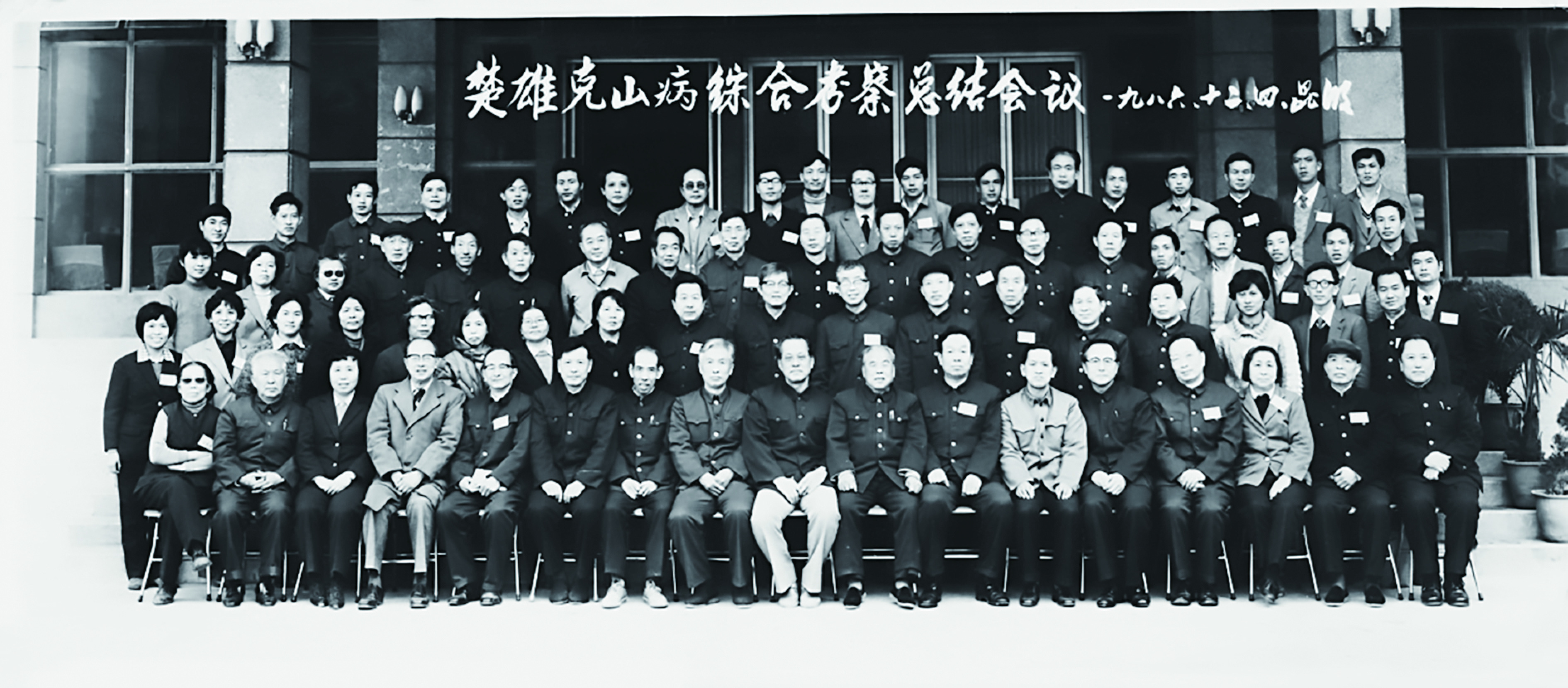
Group photo of the symposium on Keshan disease, Kunming, 1986. Front row, fourth from the right, Professor Fuyu Yang

Besides academic achievements, Professor Yang made a great contribution to academic groups, academic conferences and academic journals. He served as a director of the Biophysical Society of China, vice-president of the Chinese Society of Biochemistry and Molecular Biology, and president of the Beijing Society of Biochemistry and Molecular Biology, *etc*. In 1980, he promoted the establishment of the Biomembrane Professional Committee of the Biophysical Society of China, actively promoted the holding of the first National Membrane Biology Symposium in 1981, and served as the chairman of the first conference.

Particularly, Professor Yang devoted a lot of effort to the development of the journal *Acta Biophysica Sinica* which then evolved into the present journal *Biophysics Reports*. In 1992, he became the editor-in-chief of *Acta Biophysica Sinica,* and served in this position with a high sense of responsibility and great enthusiasm until 2003. Many young biophysicists benefited from this journal.

With great grief, we mourn the passing away of this man who made great contributions to China's biophysical and biochemical research.

## Conflict of interest

 Editorial Board of Biophysics Reports declare that they have no conflict of interest.

